# Preparedness of Hospitals in the Republic of Ireland for an Influenza Pandemic, an Infection Control Perspective

**DOI:** 10.1186/s12889-015-2025-6

**Published:** 2015-09-03

**Authors:** Mary Reidy, Fiona Ryan, Dervla Hogan, Sean Lacey, Claire Buckley

**Affiliations:** Bon Secours Hospital, Tralee, County Kerry Ireland; Department of Public Health, Cork, Ireland; Department of Epidemiology and Public Health, University College Cork, Cork, Ireland; Department of Mathematics, Cork Institute of Technology, Cork, Ireland

**Keywords:** Influenza, Pandemic, Infection control, Infection control nurse, Personal protective equipment

## Abstract

**Background:**

When an influenza pandemic occurs most of the population is susceptible and attack rates can range as high as 40–50 %. The most important failure in pandemic planning is the lack of standards or guidelines regarding what it means to be ‘prepared’. The aim of this study was to assess the preparedness of acute hospitals in the Republic of Ireland for an influenza pandemic from an infection control perspective.

**Methods:**

This was a cross sectional study involving a questionnaire completed by infection control nurses, time period from June – July 2013, (3 weeks) from acute public and private hospitals in the Republic of Ireland. A total of 46 out of 56 hospitals responded to the questionnaire.

**Results:**

From a sample of 46 Irish hospitals, it was found that Irish hospitals are not fully prepared for an influenza pandemic despite the 2009 Influenza A (H1N1) pandemic. In 2013, thirty five per cent of Irish hospitals have participated in an emergency plan or infectious disease exercise and have plans or been involved in local planning efforts to care for patients at non-health care facilities. Sixty per cent of Irish hospitals did not compile or did not know if the hospital had compiled a “lessons learned” from any exercise that were then used to revise emergency response plans. Fifty two per cent of hospitals have sufficient airborne isolation capacity to address routine needs and have an interim emergency plan to address needs during an outbreak. Fifty one percent of hospitals have taken specific measures to stockpile or have reserve medical supplies e.g. masks, ventilators and linen.

**Conclusions:**

This is the first study carried out in the Republic of Ireland investigating the current preparedness for an influenza pandemic from an infection control perspective.

Deficits exist in the provision of emergency planning committees, testing of emergency plans, airborne isolation facilities, stockpiling of personal protective equipment (PPE) and medical supplies and organisational schemes/incentives for healthcare workers to continue to work in a pandemic. While Irish standards are comparable to findings from international studies, the health care service needs to continue to enhance preparedness for an influenza pandemic and implement standard preparedness guidance for all Irish hospitals.

## Background

The historical degree of antigenic natural process and occurrence of the 2009 pandemic influenza A (H1N1) virus strains shows that mutations in the future cannot be foreseen [1]. Due to the growth in global transport and urbanization, epidemics caused by a new influenza virus are likely to spread rapidly around the world [2]. Influenza pandemics occur sporadically and most of the population is susceptible so that attack rates can range as high as 40–50 % of the population [3].

The objective of pandemic planning is to assist countries to be prepared to recognise and manage an influenza pandemic. Planning can minimise the spread of the pandemic virus, decrease cases, hospitalisations and deaths, preserve essential services and mitigate the economic and social aftermaths of a pandemic [4].

Hospital emergency management plans are a vital element of hospital preparedness for magnitude casualties and need to tackle all key risks including biologic threats such as bioterrorism, emerging infectious diseases, outbreaks and pandemics [5]. The key failure in pandemic planning is the lack of standards or guidelines regarding what it means to be ‘prepared’ [6].

International studies reported similar findings to each other regarding infectious disease disaster preparedness. Hospitals had inadequate interaction and accounting procedures, poor healthcare worker training programs and inadequate stockpiled personal protective equipment and other medical equipment e.g. ventilators [5–8]. These studies highlighted deficient 24 h infection control resources, lack of preparedness for an increase in the need for negative-pressure facilities and failure of healthcare workers (HCWs) to participate in hospital pandemic preparedness drills which involved an infectious disease scenario along with lack of prioritization strategies for distribution of restricted doses of antiviral medications [5, 9–11]. Hospitals require more preparedness for infectious disease outbreaks and hospitals have not focused on testing their plans despite the evidence that preparedness is key during a pandemic [5, 10, 12, 13]. Smaller hospitals (≤99 beds) are less prepared than larger hospitals with regards to surge capacity [14]. The consistent recommendations from these studies are that hospitals need to continue to prepare and test their preparedness for an influenza pandemic. In comparison to similar studies, these authors used different audit tools in order to assess the level of preparedness for an influenza pandemic (no audit tool the same). There is no checklist currently in Ireland for Irish hospitals to assess how prepared they are for an influenza pandemic, not included in the Irish guidance for an influenza pandemic. The Centre of Diseases Control and Prevention (CDC) has a check list which assists hospitals to assess their preparedness which is not included in the Irish pandemics preparedness as a tool to assist hospitals.

The aim of this study was to assess the preparedness of acute hospitals in the Republic of Ireland for an influenza pandemic from an infection control perspective.

## Methods

This is a cross-sectional study using a questionnaire completed by infection control nurses. The participant information sheet and a letter outlining the study requesting permission to carry out the research was sent to the Chief Executive Officer (CEO)/Manager of each hospital. An e-mail was sent to the infection prevention control nurse (IPCN) of each hospital outlining the study and objectives of same, inclusion and exclusion criteria were applied as follows: Acute public and private hospitals in the Republic of Ireland were included. Nursing Homes and Psychiatric hospitals were excluded. The list of acute public and private hospitals was compiled from the Health Service Executive web site and the Health Protective Surveillance Centre surveillance scientist who collates data for the National Clostridium Difficile Associated Disease surveillance in the Republic of Ireland.

### Questionnaire Design/Data Collection Method

There is no checklist currently in Ireland for Irish hospitals to assess how prepared they are for an influenza pandemic, not included in the Irish guidance for an influenza pandemic. The Centre for Disease Control provides a checklist in order for hospitals to assess their preparedness for an influenza pandemic. International studies were cross sectional studies using questionnaires, no questionnaire was similar. The author adapted the questionnaire by Rebmann (2009b), examining the knowledge of infection control nurses of their hospitals preparedness for an influenza pandemic using a quantitative study design. The researcher used this questionnaire for the study with permission from the author Rebmann. Prior to piloting the questionnaire, it was edited using English terminology rather than Americanized versions. The name of the hospital was sought in order for follow-up phone calls to hospitals to fill in blank answers. This questionnaire was modified and piloted by IPCNs in three hospitals. No modifications were required following their evaluation. The questionnaire included 42 questions to be answered. This questionnaire allowed hospitals to give comments on the best and worst aspects of their hospitals preparedness for an influenza pandemic which was analysed descriptively. Open ended questions were included in the questionnaire; these comments will be reviewed in further research.

The questionnaire contained 42 questions. The domains evaluated in the questionnaire included the following:

1. Pandemic emergency preparedness
2. Airborne isolation
3. Staffing
4. Vaccines administration to healthcare workers (HCW's)
5. Stockpile of supplies.

The name of each hospital was sought in order for follow-up phone calls to hospitals to fill in blank answers. The questionnaire contained a number of ‘Yes’, ‘No’ or 'Don't know' style questions and also allowed IPCNs to give comments. Consent was obtained when survey completed by each IPCN and returned to author. Time period to sending out questionnaires and receiving responses was from June – July 2013, (3 weeks) from acute public and private hospitals in the Republic of Ireland. A total of 46 out of 56 hospitals responded to the questionnaire

The statistical computer package IBM SPSS, Version 22 was used to perform quantitative analysis on the collected data. In order to perform analysis of data, it was necessary to code the response variables i.e., yes = 1, no = 0, don't know = blank. Data cleaning was carried out throughout the data entry process. Analysis of the data was performed through descriptive statistics, such as frequencies for each of the variables. Contingency tables were used to describe the relationship between question responses. A chi-squared test for association was used to determine whether or not any associations observed were statistically significant (p ≤ 0.05). Hospital size categories were selected from arbitrary, no existing hospital classification in use in Ireland. Hospital sizing involved the following:

- Small <100 beds (8 hospitals responded)
- Medium 100–199 beds (18 hospitals responded)
- Large >199 beds (20 hospitals responded)

Contingency tables were used to describe the relationship between question responses. A chi-squared test for association was used to determine whether or not any associations observed were statistically significant (p ≤ 0.05).

### Ethics

Ethical approval was granted by the Clinical Research Committee of the Cork Teaching Hospitals (public hospitals) and the Bon Secours Group Ethics Committee (private hospitals) in Ireland.

## Results

The response rate was 85 %; 46 out of 56 acute public and private hospitals completed the questionnaire. Response rate is broken down further by the following: small sized hospitals <100 beds (8 responses), medium sized hospitals 100–199 beds (18 responses) and large sized hospitals >199 beds (20 responses) see Table 1 on the break down of results.

### 1. Pandemic emergency preparedness:

Eighty five percent (n = 39) of hospitals have an emergency planning committee and of those hospitals that have a committee, seventy nine per cent include in their scope planning for an influenza pandemic (see Table 2). Smaller hospitals were significantly less likely to have an emergency planning committee (p = .045). Sixty seven per cent (n = 31) of emergency planning committees include planning for influenza pandemic (see Table 3). There was no statistical association found between whether or not emergency planning committees include planning for influenza pandemic and hospital size (p = 0.972). The highest result of seventy two per cent (n = 13) accounts for medium sized hospitals, with a similar result of sixty two per cent (n = 5) for small and sixty five per cent (n = 13) for large hospitals.

**Table 1:** Break down of results on Preparedness of Hospitals in the Republic of Ireland for an Influenza Pandemic, an Infection Control Perspective

Pandemic emergency preparedness	Small hospitals compliance	Medium hospitals compliance	Large hospitals	Total percentage compliance	Total number of hospitals
Existence of emergency planning committee	50 % (n = 4)	94 % (n = 17)	90 % (n = 18)	85 % (n = 39)	39
Hospitals with an emergency planning committee, percentage of committees which included in their scope planning for an influenza pandemic				79 %	36
Percentage of emergency planning committees which include planning for influenza pandemic	62 % (n = 5)	72 % (n = 13)	65 % (n = 13)	67 %	31
Percentage of hospitals which participated in an emergency plan or infectious disease exercise in the past 12 months	25 % (n = 2)	40 % (n = 7)	35 % (n = 16)	35 %	16
Percentage of hospitals which involve community participation when conducting an emergency plan				15 %	5
Percentage of hospitals who compiled lessons learnt from emergency exercises carried out				40 %	15
Percentage of hospitals who educated staff on revisions made to emergency response plans				36 %	16
Percentage of emergency plans which include a surge capacity plan to incorporate additional staffing resources	12.5 % (n = 1)	29 % (n = 5)	37 % (n = 7)	30 %	13
Percentage of hospitals who have plans or been involved in local planning efforts to care for patients at non-health care facilities				30 %	14
Percentage of hospitals who have incentives to encourage HCW’s to continue to come to work in the event of a major infectious disease outbreak/disaster	25 % (n = 2)	22 % (n = 4)	20 % (n = 4)	22 %	10
Airborne isolation					
Percentage of hospitals who have enough airborne isolation capabilities and capacity to meet current needs	62 % (n = 5)	50 % (n = 9)	50 % (n = 10)	52 %	24
Percentage of hospitals who have an interim emergency plan for addressing airborne isolation capacity for an outbreak of prolonged airborne-spread disease to safely house patients on an emergency temporary basis	12 % (n = 1)	72 % (n = 13)	55 % (n = 11)	54 %	25
Percentage of hospitals who have sufficient plans to safely house patients during a major airborne-spread disease outbreak	12 % (n = 1)	41 % (n = 7)	45 % (n = 9)	38 %	17
Staffing					
Percentage of hospitals who have cross trained staff to treat an influx of influenza patients	12 % (n = 1)	28 % (n = 5)	15 % (n = 3)	26 %	12
Percentage of hospitals who have cross-trained staff to provide patient care outside their routine area or speciality	12 % (n = 1)	28 % (n = 5)	15 % (n = 3)	20 %	9
Percentage of hospitals who have developed policies or procedures to provide altered standards of care during a pandemic				35 %	16
Percentage of hospitals who have plans for instituting a ‘working quarantine’ for staff				20 %	9
Vaccine administration					
Percentages of hospitals who have a plan to prioritise hospital workers to receive vaccines in the event of an infectious emergency				65 %	30
Stockpile of supplies					
Percentage of hospital who have taken measures to stockpile linen, gowns, masks and other supplies	12 % (n = 1)	72 % (n = 13)	47 % (n = 9)	51 %	23
Existence of emergency planning committee	50 % (n = 4)	94 % (n = 17)	90 % (n = 18)	85 % (n = 39)	39
Hospitals with an emergency planning committee, percentage of committees which included in their scope planning for an influenza pandemic				79 %	36
Percentage of emergency planning committees which include planning for influenza pandemic	62 % (n = 5)	72 % (n = 13)	65 % (n = 13)	67 %	31
Percentage of hospitals which participated in an emergency plan or infectious disease exercise in the past 12 months	25 % (n = 2)	40 % (n = 7)	35 % (n = 16)	35 %	16
Percentage of hospitals which involve community participation when conducting an emergency plan				15 %	5
Percentage of hospitals who compiled lessons learnt from emergency exercises carried out				40 %	15
Percentage of hospitals who educated staff on revisions made to emergency response plans				36 %	16
Percentage of emergency plans which include a surge capacity plan to incorporate additional staffing resources	12.5 % (n = 1)	29 % (n = 5)	37 % (n = 7)	30 %	13
Percentage of hospitals who have plans or been involved in local planning efforts to care for patients at non-health care facilities				30 %	14
Percentage of hospitals who have incentives to encourage HCW’s to continue to come to work in the event of a major infectious disease outbreak/disaster	25 % (n = 2)	22 % (n = 4)	20 % (n = 4)	22 %	10
Airborne isolation					
Percentage of hospitals who have enough airborne isolation capabilities and capacity to meet current needs	62 % (n = 5)	50 % (n = 9)	50 % (n = 10)	52 %	24
Percentage of hospitals who have an interim emergency plan for addressing airborne isolation capacity for an outbreak of prolonged airborne-spread disease to safely house patients on an emergency temporary basis	12 % (n = 1)	72 % (n = 13)	55 % (n = 11)	54 %	25
Percentage of hospitals who have sufficient plans to safely house patients during a major airborne-spread disease outbreak	12 % (n = 1)	41 % (n = 7)	45 % (n = 9)	38 %	17
Staffing					
Percentage of hospitals who have cross trained staff to treat an influx of influenza patients	12 % (n = 1)	28 % (n = 5)	15 % (n = 3)	26 %	12
Percentage of hospitals who have cross-trained staff to provide patient care outside their routine area or speciality	12 % (n = 1)	28 % (n = 5)	15 % (n = 3)	20 %	9
Percentage of hospitals who have developed policies or procedures to provide altered standards of care during a pandemic				35 %	16
Percentage of hospitals who have plans for instituting a ‘working quarantine’ for staff				20 %	9
Vaccine administration					
Percentages of hospitals who have a plan to prioritise hospital workers to receive vaccines in the event of an infectious emergency				65 %	30
Stockpile of supplies					
Percentage of hospital who have taken measures to stockpile linen, gowns, masks and other supplies	12 % (n = 1)	72 % (n = 13)	47 % (n = 9)	51 %	23

**Table 2:** Contingency table of hospital size with emergency planning committee

		Does the hospital have an emergency planning committee?			Total
		Yes	No	Don't know	
Hospital size	<100	4 (50.0 %)	3 (37.5 %)	1 (12.5 %)	8 (100.0 %)
	100-199	17 (94.4 %)	1 (5.6 %)	0 (0.0 %)	18 (100.0 %)
	>199	18 (90.0 %)	1 (5.0 %)	1 (5.0 %)	20 (100.0 %)
Total		39 (84.8 %)	5 (10.9 %)	2 (4.3 %)	46 (100.0 %)

**Table 3:** Contingency table of hospital size with planning for influenza pandemic

		Does emergency planning committees include planning for influenza pandemic?			Total
		Yes	No	Don't know	
Hospital size	<100	5 (62.5 %)	2 (25.0 %)	1 (12.5 %)	8 (100.0 %)
	100-199	13 (72.2 %)	4 (22.2 %)	1 (5.6 %)	18 (100.0 %)
	>199	13 (65.0 %)	5 (25.0 %)	2 (10.0 %)	20 (100.0 %)
Total		31 (67.4 %)	11 (23.9 %)	4 (8.7 %)	46 (100.0 %)

Thirty five per cent (n = 16) of hospitals have participated in an emergency plan or infectious disease exercise in the past twelve months (see Table 4). The majority of hospitals (56.5 %) (n = 26) have not participated in an emergency plan or infectious disease exercise in the last 12 months (p < 0.001). A minority (17.6 % (n = 6)) of hospitals involved community participation when conducting an emergency plan (p = 0.037).

**Table 4:** Contingency table of hospital participation

Response	Participated in an emergency plan or infectious disease exercise in the past 12 months	Community participation	Compiled lessons learned	Educated/trained on revisions	Emergency plan include a surge capacity plan
Yes	16 (34.8 %)	6 (17.6 %)	16 (41.0 %)	16 (35.6 %)	13 (29.5 %)
No	26 (56.5 %)	10 (29.4 %)	15 (38.5 %)	21 (46.7 %)	18 (40.9 %)
Don't know	4 (8.7 %)	18 (52.9 %)	8 (20.5 %)	8 (17.8 %)	13 (29.5 %)
Total	46 (100.0 %)	34 (100.0 %)	39 (100.0 %)	45 (100.0 %)	44 (100.0 %)
p-value	<0.001	0.037	0.232	0.057	0.567

Fifteen per cent (n = 5) of hospitals involved community participation when conducting an emergency plan. Forty per cent (n = 15) of hospitals compiled lessons learned from emergency exercises carried out. Thirty six per cent (n = 16) of hospitals educated staff on revisions made to emergency response plans. Thirty per cent (n = 13) of emergency plans included a surge capacity plan to incorporate additional staffing resources.

Only thirty per cent (n = 14) of hospitals have plans or been involved in local planning efforts to care for patients at non-health care facilities (p = 0.001, see Table 5). While twenty two per cent (n = 10) of hospitals have incentives to encourage HCWs to continue to come to work in the event of a major infectious disease outbreak/disaster (p < 0.001).

**Table 5:** Contingency table of hospitals involved in local planning and staff incentives

Response	Established plans or been involved in local planning efforts to care for a patients at non-health care facilities	Incentives to encourage HCWs to continue to come to work in the event of a major infectious disease outbreak/disaster
Yes	14 (30.4 %)	10 (21.7 %)
No	26 (56.5 %)	30 (65.2 %)
Don't know	6 (13.0 %)	6 (13.0 %)
Total	46 (100.0 %)	46 (100.0 %)
p-value	0.001	<0.001

### 2. Airborne isolation capabilities

From Table 6, fifty two per cent (n = 24) of hospitals have enough airborne isolation capabilities and capacity (in terms of rooms or areas) to meet their current routine needs.

**Table 6:** Contingency table of hospitals having sufficient and preparation for isolation

	Enough airborne isolation capabilities and capacity	Interim emergency plan for addressing airborne isolation capacity
Yes	24 (52.2 %)	25 (54.3 %)
No	22 (47.8 %)	14 (30.4 %)
Don't know	0 (0.0 %)	7 (15.2 %)
Total	46 (100.0 %)	46 (100.0 %)
p-value	0.883	0.005

Fifty four per cent (n = 25) of hospitals have an interim emergency plan for addressing airborne isolation capacity for an outbreak of prolonged airborne-spread disease to safely house patients on an emergency temporary basis. From Table 7, twelve and a half per cent (n = 1) of smaller hospitals and fifty five per cent (n = 11) of larger sized hospitals have an interim emergency plan compared to seventy two percent (n = 13) of medium sized hospitals.

**Table 7:** Contingency table of hospital size and interim emergency airborne isolation capacity

		Interim emergency plan for addressing airborne isolation capacity			Total
		Yes	No	Don't know	
Hospital size	<100	1 (12.5 %)	5 (62.5 %)	2 (25.0 %)	8 (100.0 %)
	100-199	13 (72.2 %)	4 (22.2 %)	1 (5.6 %)	18 (100.0 %)
	>199	11 (55.0 %)	5 (25.0 %)	4 (20.0 %)	20 (100.0 %)
Total		25 (54.3 %)	14 (30.4 %)	7 (15.2 %)	46 (100.0 %)

Thirty eight per cent (n = 17) of hospitals have sufficient plans to safely house patients during a major airborne-spread disease outbreak (Table 8, p = 0.074).

**Table 8:** Contingency table to house patients during major airborne disease outbreak

Sufficient plans to safely house patients during a major airborne-spread disease outbreak	Frequency	Percent
Yes	17	37.8
No	20	44.4
Don't know	8	17.8
Total	45	100.0

### 3. Staffing

Low levels of cross-training (twenty six percent, (n = 12)) to treat an influx of influenza patients was reported overall (see Table 9, p < 0.001).

**Table 9:** Contingency table re staffing preparedness

Response	Cross-training to treat an influx of influenza patients	Cross-trained staff to provide patient care outside their routine area	Plans for having designated staff limited to treat either influenza or non-influenza patients	Developed policies/procedures	Plans for instituting "working quarantine" for staff
Yes	12 (26.1 %)	9 (19.6 %)	23 (50.0 %)	16 (34.8 %)	10 (21.7 %)
No	30 (65.2 %)	31 (67.4 %)	16 (34.8 %)	24 (52.2 %)	27 (57.7 %)
Don't know	4 (8.7 %)	6 (13.0 %)	7 (15.2 %)	6 (13.0 %)	9 (19.6 %)
Total	46 (100.0 %)	46 (100.0 %)	46 (100.0 %)	46 (100.0 %)	46 (100.0 %)
p-value	<0.001	<0.001	0.015	0.005	0.001

Twenty per cent of (n = 9) hospitals have cross-trained staff to provide patient care outside their routine area or speciality (Table 9, p < 0.001). Fifty per cent (n = 25) of hospitals have plans during a pandemic influenza outbreak to have designated staff limited to treat either influenza or non-influenza patients (Table 9, p = 0.015). Thirty five per cent (n = 13) of hospitals have developed policies or procedures to provide altered standards of care during a pandemic (Table 9, p = 0.005).

Twenty per cent (n = 16) of hospitals have plans for instituting a “working quarantine” for staff (Table 9, p < 0.001). All hospitals surveyed had an infection control nurse and the average number of nurses by weighted time equivalent per hospital was 1.78. Seventy six per cent of hospitals have an infection control professional available for immediate (within fifteen minutes) verbal consultation.

### 4. Vaccine administration

Sixty five per cent (n = 30) of hospitals have a plan to prioritise hospital workers to receive vaccines in the event of an infectious emergency (Table 10, p < 0.001).

**Table 10:** Contingency table vaccines and healthcare workers

Response	Prioritise hospital workers to receive vaccines in the event of an infectious emergency	Included HCWs family members to be part of the hospitals prioritisation plan
Yes	30 (65.2 %)	2 (5.7 %)
No	7 (15.2 %)	27 (77.1 %)
Don't know	9 (19.6 %)	6 (17.1 %)
Total	46 (100.0 %)	35 (100.0 %)
p-value	<0.001	<0.001

**Table 11:** Contingency table of hospital size with measures of stockpile

		Measures to stockpile			Total
		Yes	No	Don't know	
Hospital size	<100	1 (12.5 %)	5 (62.5 %)	2 (25.0 %)	8 (100.0 %)
	100-199	13 (72.2 %)	5 (27.8 %)	0 (0.0 %)	18 (100.0 %)
	>199	9 (47.4 %)	9 (47.4 %)	1 (5.3 %)	19 (100.0 %)
Total		23 (51.1 %)	19 (42.2 %)	3 (6.7 %)	45 (100.0 %)

Only two hospitals included HCWs family members to be part of the hospitals prioritisation plan for receiving vaccine or anti-infective therapy in the event of an infectious disease emergency Table 10, p <0.001).

#### 5. Stockpile of supplies

Fifty one per cent (n = 23) of hospitals have taken measures to stockpile linen, gowns, masks and other supplies, (see Table 11).

## Discussion

The main findings from this study showed hospitals are not fully prepared from an infection control perspective for an influenza pandemic.

### 1. Pandemic emergency preparedness

Our results revealed 85 % of Irish hospitals had an emergency planning committee and 67 % of such committees included planning for an influenza pandemic. There is a statistically significant association between hospital size and the existence of an emergency planning committee i.e. 50 % of smaller hospitals (<100 beds) have emergency planning committees compared to >90 % of medium and larger hospitals surveyed. Chinese findings were similar [12] where 85 % of hospitals had an emergency planning committee while 79 % included in their scope planning for an influenza pandemic in comparison to findings from a Chinese study which demonstrated a higher result of 93 %.

One third of Irish hospitals had participated in an emergency plan or infectious disease exercise in the last year in comparison to Canadian findings [13] which showed a higher result of 84.5 % of hospitals had tested their plans while Chinese studies [12] demonstrated 55 % of hospitals had evaluated and reviewed their emergency plan at least once and a similar result of 84 % of carrying out an infectious disease scenario in the previous year [14].

Irish hospitals demonstrated a low level of including community participation of 15 % in comparison to a rate of 86.5 % from America findings [14].

Noteworthy that only 60 % of respondents did not compile or did not know if the hospital had compiled a “lessons learned” from any exercise that were then used to revise emergency response plans while 85 % of American hospitals had compiled lessons learned from the exercise that was used to revise their emergency plan [14]. Hospital emergency plans should be updated regularly based on lessons learned from exercises [8].

### 2. Airborne isolation Capabilities

Over half of Irish hospitals had enough airborne isolation capabilities and capacity to meet the current routine needs (one per 150 acute inpatient beds, or one per 75 acute inpatient beds for regional or tertiary hospitals SARI 2009) compared to 85 % of American hospitals reported that their hospital has sufficient numbers of negative-pressure rooms to accommodate their current isolation needs [14]. Only 55 % of Irish hospitals have an interim emergency plan to address needs during an outbreak.

Significantly, only 38 % of Irish hospitals have sufficient plans to safely house patients during a major airborne disease outbreak and 54 % had an interim emergency plan for addressing airborne isolation capacity for an outbreak of prolonged airborne -spread disease to safely house patients on an emergency temporary basis [14].

### 3. Staffing

Only 26 % of Irish hospitals have cross-trained staff to be able to treat an influx of influenza patients while 22 % have cross-trained staff to provide patient care outside their routine area or speciality compared to a similar study of 24.6 % showing similar results [14].

### 4. Vaccines

Sixty-five percent of Irish hospitals have a plan to prioritise hospital workers to receive vaccines in the event of an infectious emergency. In regards to international studies there is no comparison to vaccines. This low reported percentage may be due to IPCNs not having the information as occupational health nurses would normally be responsible for vaccines.

### 5. Stockpile of supplies

The emergency plan should include protocols for stockpiling or obtaining additional staff, medical and laboratory equipment supplies [14] whilst stockpiling a sufficient amount of PPE could be a large financial burden on each hospital; this must occur before pandemics escalate due to the inevitable shortage of PPE [15]. There is a statistically significant association between hospital size and taking measures to stockpile or have reserve medical supplies (p = 0.026). The Health Service holds a stockpile of supplies which is given to hospitals during a pandemic. Medium sized hospitals are better prepared than smaller and larger sized hospitals.

Less than half of hospitals have stockpiled or worked with the HSE to establish a stockpile of FFP2/FFP3 respirators (average of 6 days) compared to 67 % of American hospitals stockpiling N95 respirators [14].

One third of hospitals have stockpiled surgical masks (average of 5.1 days supply) compared to 46 % of American findings [14].

One fifth of hospitals have stockpiled or worked with the state to establish a stockpile of linen (average of 2.4 days supply of linen) compared to a demonstration of a higher rate of half of US hospitals indicated they had enough linen for <5 days [14].

Overall these findings are evidence for the need for greater prioritisation of pandemic preparedness stockpiling.

### Strengths of study

Forty six hospitals out of a total of 56 acute public and private hospitals in the Republic of Ireland completed the questionnaire yielding a response rate of 82 %. This was a good response rate in comparison to the literature review studies; ranging from 20 % [14] – 88.2 % [13].

### Limitations

There may have been responder bias as IPCNs completed the survey, may have focussed on strengths. Standardised validated questionnaire was not used however, previously used questionnaires were adapted and piloted prior to use. Information was limited by IPCN's as some had the information themselves due to being a member of the emergency committee, some IPCN's went to lead persons to gain answers to the questionnaire while others did not have all the information. Ten hospitals did not respond, all private hospitals responded. No reason given for not responding. Analysis was not done in regards to levels of preparedness by private and public hospitals. The question was not posed if hospitals were using a checklist in order to assess their preparedness e.g. Centre for Disease Control guidance.

## Conclusion

This is the first study in the Republic of Ireland investigating preparedness of Irish acute hospitals for an influenza pandemic from an infection control perspective, acute hospitals in the Republic of Ireland have deficits regarding emergency planning committees, testing of emergency plans, airborne isolation facilities and incentives to encourage HCW’s to come to work in line with the literature. The Republic of Ireland needs to address gaps in influenza pandemic preparedness.

### Recommendations

Hospitals should prepare and regularly test their preparedness for an influenza pandemic using the standardised recognised tools.

There should be national agreement on the use of a standardised internationally recognised tool to be used in all acute hospitals for this purpose.

The overarching recommendation is the need for a strategic approach to pandemic preparedness and elements of this research could be used to inform health policy in the Republic of Ireland.

## Abbreviations

APIC: Association for Professionals in Infection Control and Epidemiology
CDC: Centre for Disease Control
CI: Confidence Interval
DH: Department Health
DOH: Department of Health
GAR: Global Assessment Report
HCP: Health Care Professionals
HCW: Health Care Worker
HSE: Health Service Executive
IC: Infection Control
ICP: Infection Control Preventionist
ICN: Infection Control Nurse
IPCN: Infection Prevention and Control Nurse
IP: Infection Prevention
PHE: Public Health Emergency
PPE: Personal Protective Equipment
SARS: Severe Acute Respiratory Syndrome
SPSS: Statistical Package for the Social Sciences
US: United States
WHO: World Health Organisation
